# The Language Network Reliably “Tracks” Naturalistic Meaningful Nonverbal Stimuli

**DOI:** 10.1162/nol_a_00135

**Published:** 2024-06-03

**Authors:** Yotaro Sueoka, Alexander Paunov, Alyx Tanner, Idan A. Blank, Anna Ivanova, Evelina Fedorenko

**Affiliations:** Department of Brain and Cognitive Sciences, Massachusetts Instititute of Technology, Cambridge, MA, USA; Department of Neuroscience, Johns Hopkins University, Baltimore, MD, USA; McGovern Institute for Brain Research, Massachusetts Instititute of Technology, Cambridge, MA, USA; Cognitive Neuroimaging Unit, INSERM, CEA, CNRS, Université Paris-Saclay, NeuroSpin center, Gif/Yvette, France; Department of Psychology and Linguistics, University of California Los Angeles, Los Angeles, CA, USA; School of Psychology, Georgia Institute of Technology, Atlanta, GA, USA; Program in Speech and Hearing Biosciences and Technology, Harvard University, Cambridge, MA, USA

**Keywords:** fMRI, intersubject correlation, language network, naturalistic stimuli, nonverbal semantics

## Abstract

The language network, comprised of brain regions in the left frontal and temporal cortex, responds robustly and reliably during language comprehension but shows little or no response during many nonlinguistic cognitive tasks (e.g., Fedorenko & Blank, 2020). However, one domain whose relationship with language remains debated is semantics—our conceptual knowledge of the world. Given that the language network responds strongly to meaningful linguistic stimuli, could some of this response be driven by the presence of rich conceptual representations encoded in linguistic inputs? In this study, we used a naturalistic cognition paradigm to test whether the cognitive and neural resources that are responsible for language processing are also recruited for processing semantically rich nonverbal stimuli. To do so, we measured BOLD responses to a set of ∼5-minute-long video and audio clips that consisted of meaningful event sequences but did not contain any linguistic content. We then used the intersubject correlation (ISC) approach (Hasson et al., 2004) to examine the extent to which the language network “tracks” these stimuli, that is, exhibits stimulus-related variation. Across all the regions of the language network, meaningful nonverbal stimuli elicited reliable ISCs. These ISCs were higher than the ISCs elicited by semantically impoverished nonverbal stimuli (e.g., a music clip), but substantially lower than the ISCs elicited by linguistic stimuli. Our results complement earlier findings from controlled experiments (e.g., Ivanova et al., 2021) in providing further evidence that the language network shows some sensitivity to semantic content in nonverbal stimuli.

## INTRODUCTION

A set of brain regions in the left frontal and temporal cortex in the human brain respond robustly and reliably during language comprehension, both auditory and visual (e.g., Fedorenko et al., 2010; Silbert et al., 2014; Vagharchakian et al., 2012). In brain imaging studies, these regions exhibit highly selective responses to language processing over diverse cognitive tasks, including those that bear parallels to language and have long been argued to share resources with language. For example, language-responsive regions show little or no response to arithmetic (e.g., Amalric & Dehaene, 2019; Fedorenko et al., 2011; Monti et al., 2012), music (e.g., Chen et al., 2023; Fedorenko et al., 2011; Rogalsky et al., 2011), logic (e.g., Monti et al., 2009), executive control (e.g., Fedorenko et al., 2011; Mineroff et al., 2018), action/gesture observation (e.g., Jouravlev et al., 2019; Pritchett et al., 2018), theory of mind (e.g., Deen et al., 2015; Paunov, 2018; Paunov et al., 2022; Shain, Paunov, et al., 2023), and even the processing of computer languages (e.g., Ivanova et al., 2020; Liu et al., 2020). Evidence from patients with aphasia converges with these findings, showing that the loss of language production and comprehension abilities can leave other cognitive abilities unimpaired (e.g., Apperly et al., 2006; Bek et al., 2010; Benn et al., 2023; Lecours & Joanette, 1980; Varley et al., 2001, 2005; Varley & Siegal, 2000; Willems et al., 2011; see Fedorenko & Varley, 2016, for a review).

However, one domain whose relationship with language remains debated is semantic knowledge—generalized, abstract conceptual information about objects/entities, actions, and events, both verbal and nonverbal (Ivanova et al., 2021; Lambon Ralph et al., 2017; Patterson et al., 2007; Warrington, 1975). On the one hand, language understanding is inextricably linked to semantics. Whether producing or comprehending a sentence, accessing the meaning of words and constructions is an essential component of linguistic processing (e.g., Goldberg, 2010; Katz & Fodor, 1963; Pustejovsky, 2005). On the other hand, semantic knowledge is not restricted to language: nonverbal stimuli, such as pictures and sounds, can also evoke rich conceptual representations and support reasoning about the world. Here, we ask: Is the language network—or any of its components—sensitive to semantic knowledge regardless of whether or not it is delivered linguistically?

Previous work provides conflicting evidence about the role of the language network in nonverbal semantic processing. A number of brain imaging investigations have reported overlapping activations in the lateral frontal and temporal brain areas for the processing of words and object pictures (e.g., Devereux et al., 2013; Handjaras et al., 2017; Visser et al., 2012), words and actions (Wurm & Caramazza, 2019), or sentences and event pictures (e.g., Ivanova et al., 2021), although the components of the language network that showed overlap differ across studies. For example, Visser et al. (2012) and Ivanova et al. (2021) reported overlap between verbal and nonverbal semantic processing across the language network, but Devereux et al. (2013) did not find overlap in the anterior temporal cortex, and Wurm and Caramazza (2019) reported overlap in the posterior temporal cortex, but not in the frontal areas. Furthermore, neuropsychological evidence from individuals with aphasia indicates that linguistic and semantic processing are neurally dissociable, such that semantic processing can appear intact in the presence of even severe linguistic deficits (e.g., Chertkow et al., 1997; Colvin et al., 2019; Ivanova et al., 2021; Saygin et al., 2004; Warren & Dickey, 2021; Whitehouse et al., 1978).

Most past brain imaging studies that have reported overlap between responses to verbal and semantically meaningful nonverbal (typically, pictorial) stimuli have relied on traditional task-based paradigms. In such paradigms, participants read words or view pictures and perform a semantic task on those words/pictures. One possible explanation of the observed overlap is that language regions respond during the processing of pictures because participants activate the corresponding verbal labels as they come up with the response to the (often verbally presented) task prompt (e.g., activating the word “hammer” when processing a picture of a hammer and deciding its semantic category; Devereux et al., 2013). Activating the label may help hold the concept in working memory or retrieve relevant semantic knowledge but not be essential to semantic processing per se (see also Benn et al., 2023, for discussion).

In this study, we aimed to determine whether the language network responds to semantically meaningful nonlinguistic stimuli in the absence of an explicit task. To do so, we turned to naturalistic stimuli (Hasson et al., 2018) and leveraged the intersubject correlation (ISC) approach (Hasson et al., 2004; Hasson et al., 2008). In this approach, the similarity of neural fluctuations during some naturalistic stimulus is computed across participants, and the degree of intersubject similarity is taken to reflect the degree to which a given voxel or brain region responds to features of the relevant stimulus (or “tracks” the stimulus).

The advantages of naturalistic meaningful stimuli that unfold over time in a task-free setting are fourfold. First, such stimuli provide a good approximation to many real-life scenarios and can thus yield important insights about the role of the language network in semantic processing. Second, because rich naturalistic stimuli are generally engaging and enjoyable, they help maintain the participants’ attention even in the absence of an explicit task. Third, the use of naturalistic stimuli likely reduces task-related verbal recoding that may occur during traditional task-based designs (Greene & Fei-Fei, 2014; Trueswell & Papafragou, 2010). And finally, by continuously capturing fluctuation in stimulus tracking patterns, the ISC approach allows distinguishing *consistent* brain region engagement from engagement that takes place only *occasionally* (e.g., during parts of the stimulus where verbal recoding may be likely to happen). Consistent engagement would be reflected in ISCs that are not strongly affected by the removal of response peaks, whereas occasional engagement would be reflected in ISCs that are primarily driven by the occasional peaks (points of strong responses) and therefore substantially weakened by their removal.

Our critical stimuli consisted of ∼5-minute-long video and audio clips that were rich in semantic content (defined as described below) but did not contain any language. For comparison, we also included naturalistic linguistic stimuli, which have been previously shown to elicit high ISCs in the language network (e.g., Blank & Fedorenko, 2017; Fedorenko & Blank, 2020; Lerner et al., 2011; Paunov et al., 2022; Wilson et al., 2008), and semantically impoverished nonverbal stimuli, like music, which serve as negative controls. For simplicity, we refer to semantically rich stimuli as *meaningful*, or +M and semantically impoverished stimuli as *non-meaningful*, or −M.

The key operational criteria we used to select stimuli with rich semantic content included the presence of (a) objects/entities/people, actions, and events (e.g., “bird” depicted visually, “chasing” depicted visually, “brushing teeth” conveyed auditorily with a prototypical sound), and (b) a storyline, such that the sequences of events are sensible (e.g., a man falls asleep at church and rolls off the pew, or a sound of an alarm clock is followed by a sound of a yawn) and interpretation would become more difficult if the event order was shuffled. The latter criterion is another feature that distinguishes our study from most prior neuroimaging studies examining verbal/nonverbal semantic processing (cf. Baldassano et al., 2018; Thierry & Price, 2006), where participants typically view a series of discrete temporally unconnected stimuli.

Our operational criteria for rich semantic content/stimulus meaningfulness raise the question of the relationship between *meaning* in general and *socially relevant meanings*/*social reasoning*. At a minimum, any story-like sequence of events, even if devoid of social content, may invoke reasoning about the mental states of characters (and/or the implicit storyteller) in order to understand the relationships between events (e.g., a character’s actions, even if nonsocial in nature, typically depend on what they want, think, or believe). In practice, most noncontrived story-like event sequences are social in a richer sense: They involve actions performed by or happening to social agents, and often social interactions. Indeed, three of our four meaningful nonverbal stimuli include explicit social content, and we have previously used the data from those stimuli (as well as from the linguistic stimuli) in another study to probe the theory of mind network’s and the language network’s sensitivity to mental state content (Paunov et al., 2022; see Table S1 in the Supporting Information, available at https://doi.org/10.1162/nol_a_00135, for overlap and differences across the two datasets). However, the research question addressed in the current study is conceptually distinct, focusing on the language network’s engagement with semantically meaningful stimuli more broadly. We return to the relationship between meaningfulness and social information as they relate to the language network in the Discussion.

To foreshadow the key results, we found that the language network shows significantly higher ISCs during the processing of meaningful nonverbal stimuli compared to non-meaningful nonverbal stimuli. These ISCs were nonetheless lower than those elicited during the processing of linguistic stimuli, in line with abundant evidence that linguistic stimuli are the preferred stimulus class for the language network. Our findings suggest that the language network is recruited for the processing of semantically rich inputs even in the absence of language, although this engagement is lower than when processing linguistic meaning.

## MATERIALS AND METHODS

### General Approach

To investigate whether the language brain regions track semantic content in nonverbal stimuli, we combined two methodologies commonly used in neuroimaging research (similar to, e.g., Blank & Fedorenko, 2017): functional localization (e.g., Brett et al., 2002; Fedorenko et al., 2010; Saxe et al., 2006) and rich naturalistic stimuli analyzed with ISC (Hasson et al., 2004; Hasson et al., 2008). In this section, we motivate these methodological choices.

First, to account for interindividual variability in the location of the language network (e.g., Amunts et al., 1999; Fedorenko & Blank, 2020; Fedorenko et al., 2010; Lipkin et al., 2022; Tomaiuolo et al., 1999), we defined language regions in each participant individually using a well-established functional localizer task (Fedorenko et al., 2010). This approach yields greater sensitivity, functional resolution, and interpretability compared to the traditional group-averaging approach, where functional correspondence across participants is assumed to hold voxel-wise (e.g., Fedorenko, 2021; Nieto-Castañón & Fedorenko, 2012).

Second, naturalistic stimuli are more ecologically valid and thus more likely to elicit neural responses that reflect how the target brain region or network is recruited during real-life cognitive processing. However, the less constrained nature of the stimuli poses a challenge when interpreting the observed blood oxygen-level dependent (BOLD) signal, namely, that there are no well-defined conditions within a stimulus from which to construct model-based predictors of neural activity over time. Here, we rely on ISCs, a method introduced by Hasson et al. (2004) to tackle this issue. The reasoning behind the ISC approach is as follows: If a voxel or a brain region tracks certain features of a stimulus, then the BOLD signal should be time locked to the appearance of those features in the stimulus, which would be reflected in similar patterns of fluctuations across participants (i.e., reliable ISCs). Thus, the average time course across participants serves as the model for a given left-out participant.

In the next sections, we detail each analysis method and describe other aspects of the study’s design and procedure.

### Participants

**Figure 1.**
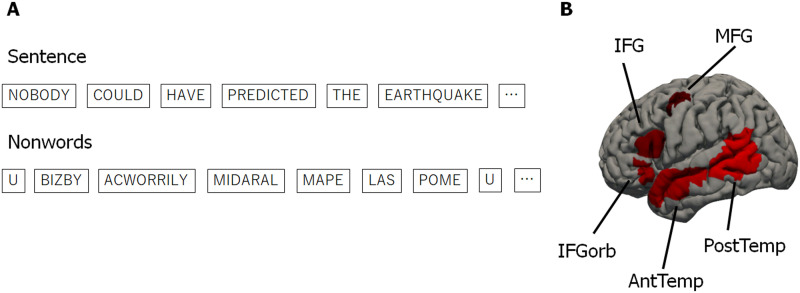
Language localizer task. A. An example sentence/nonword sequence presented during the language localizer task. Words/nonwords were presented one at a time for 450 ms each. B. Five language parcels that were used to define the language network for each individual. IFGorb = inferior frontal gyrus, orbital portion; IFG = inferior frontal gyrus; MFG = middle frontal gyrus; AntTemp = anterior temporal lobe; PostTemp = posterior temporal lobe. Each participant’s functional regions of interest were defined as the top 10% of most language-responsive voxels within each parcel.

Forty-nine native English speakers between the ages of 19 and 48, recruited from the Massachusetts Institute of Technology (MIT) and the surrounding community, were paid for their participation. Two participants were removed from the analysis due to poor quality of the functional localizer data, with the exclusion criterion defined as fewer than 100 suprathreshold voxels (at the *p* < 0.001 uncorrected whole-brain threshold) across the language network’s parcels (Figure 1B). Of the remaining 47 participants (mean age = 24.4, *SD* = 5.08; 29 females), seven participants were left-handed (based on the Edinburgh Handedness Inventory; Oldfield, 1971), but all of those left-handed participants had a left-lateralized language network (for motivation to include left-handers in cognitive neuroscience research, see Willems et al., 2014). All participants gave informed written consent in accordance with the requirements of MIT’s Committee on the Use of Humans as Experimental Subjects (COUHES).

### Design and Procedure

Each participant completed a language localizer and a subset of the naturalistic stimuli from the critical experiment. As shown in Table 1, the majority of the participants (*n* = 27, 57%) watched/heard 8 of the 10 stimuli; one participant, all 10 stimuli; and the remaining 19 participants, between 1 and 7 stimuli due to scan duration constraints (the differences in the number of stimuli are accounted for in the analysis, as described in the Critical Analysis and Content Unit Analysis sections). Each stimulus was presented to between 14 and 34 participants, and each +M stimulus was presented to at least 29 participants (Table 2). Of the 47 participants, 40 completed the language localizer in the same session as the critical experiment, and the remaining 7 participants completed it in an earlier session (see Lipkin et al., 2023; Mahowald & Fedorenko, 2016) for evidence of high across-session reliability of localizer activations; see also Braga et al., 2020). Some participants completed additional experiments for unrelated studies.

**Table 1.** Number of participants who watched/heard each subset of naturalistic conditions.

Number of stimuli	Number of participants
1 of 10	7
2 of 10	6
3 of 10	1
4 of 10	1
6 of 10	1
7 of 10	3
8 of 10	27
all 10	1

**Table 2.** Details of the naturalistic conditions.

Condition type	Condition	Duration	*N*
+M/−L	AnimShort	5 min 48 s	33
	SilentFilm	6 min 10 s	32
	IntentShapes	4 min 50 s	32
	SoundEffectStory	4 min 36 s	31
+M/+L	Story	5 min 16 s	34
	AudioPlay	6 min 14 s	31
	Dialog	5 min 35 s	29
	ExpoText	7 min 6 s	29
−M/−L	Flute	5 min 44 s	14
	Kaleidoscope	5 min 48 s	15

*Note*. Each condition belonged to one of the three condition types. The duration of each stimulus (Duration), as well as the number of participants who watched/listened to each stimulus (*N*) are shown. The durations include 16 s long fixations at the beginning and the end of the scan (32 s total). +M/−L = +Meaning/−Language; +M/+L = +Meaning/+Language; −M/−L = −Meaning/−Language.

#### Language localizer

Participants read sentences (e.g., “Nobody could have predicted the earthquake in this part of the country”) and lists of unconnected, pronounceable nonwords (e.g., “U bizby acworrily midaral mape las pome u trint weps wibron puz”) in a blocked design (Figure 1A). Each stimulus consisted of 12 words/nonwords. For details of how the language materials were constructed, see Fedorenko et al. (2010). The materials are available at https://evlab.mit.edu/funcloc/. The sentences > nonword-lists contrast has been previously shown to reliably activate left-lateralized frontal and temporal language processing regions and to be robust to changes in the materials, task, and modality of presentation (Fedorenko et al., 2010; Mahowald & Fedorenko, 2016; Scott et al., 2017). Stimuli were presented in the center of the screen, one word/nonword at a time, at the rate of 450 ms per word/nonword. Each stimulus was preceded by a 100 ms blank screen and followed by a 400 ms screen showing a picture of a finger pressing a button, and a blank screen for another 100 ms, for a total trial duration of 6 s. Participants were asked to press a button whenever they saw the picture of a finger pressing a button. This task was included to help participants stay alert and awake. Condition order was counterbalanced across runs. Experimental blocks lasted 18 s (with 3 trials per block), and fixation blocks lasted 14 s. Each run (consisting of 5 fixation blocks and 16 experimental blocks) lasted 358 s. Each participant completed 2 runs.

#### Critical experiment

**Figure 2.**
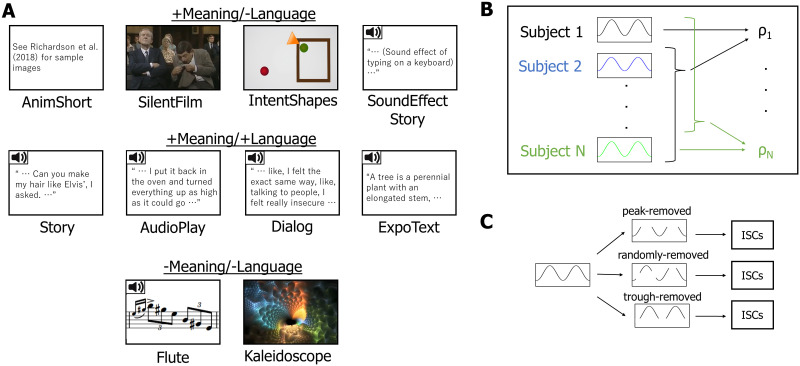
Critical task and schematics of analysis. A. Ten conditions used in the critical task. The speaker icons represent auditory stimuli; all other stimuli are visual. B. Schematics of ISC calculation. Intersubject correlations (ISCs) were computed by correlating each participant’s blood oxygen level dependent (BOLD) time series for a particular condition in a particular functional region of interest (fROI) with the average of the other participants’ BOLD time series for that condition in that fROI and Fisher-transforming the correlation coefficients. These ISCs were used for analysis with linear mixed-effects models. C. Schematics for the peak-removal analysis. From each time course, peak, trough, or random time points were removed. These trimmed time courses were then used to compute the ISCs as shown in B.

Participants passively watched or listened to a set of naturalistic stimuli (Figure 2A). Auditory stimuli were presented over scanner-safe Sensimetrics headphones (Sensimetrics, 2023). Each of 10 stimuli (conditions) belonged to one of three condition types (Figure 2): +Meaning/−Language (+M/−L), +Meaning/+Language (+M/+L), and −Meaning/−Language (−M/−L). The stimuli within each condition type were chosen to vary in their presentation modalities (visual/auditory) and styles (e.g., for visual stimuli: live action/animated movie/abstract shapes) to ensure generalizability within a condition type. The four stimuli from the critical condition were rich in semantic content but did not contain any language (+M/−L): (i) an animated short film (“Partly Cloudy”; Sohn, 2009; denoted as “AnimShort” hereafter); (ii) a clip from a live action television show (*Mr. Bean*; Davies, 1992; denoted as “SilentFilm” hereafter), (iii) a custom-created Heider and Simmel-style animation (Heider & Simmel, 1944) consisting of simple geometric shapes moving in ways as to suggest intentional interactions designed to tell a story (e.g., a shape gets locked up inside a space, another shape goes on a quest to get help to release it), denoted as “IntentShapes” hereafter; and (iv) a custom-created audio story consisting of a series of sounds that represents common events in a typical morning, denoted as “SoundEffectStory” hereafter. Four stimuli from the positive control condition used semantically rich verbal materials (+M/+L): (i) a story (“Elvis” from the Natural Stories corpus; Futrell et al., 2021; denoted as “Story” hereafter); (ii) an audio play (an audio segment from an HBO miniseries, *Angels in America*; Kushner & Nichols, 2003; denoted as “AudioPlay” hereafter); (iii) a naturalistic dialog (a casual unscripted conversation between two female friends), denoted as “Dialog” hereafter; and (iv) an expository text (a text about trees adapted from Wikipedia; “Tree,” 2024; denoted as “ExpoText” hereafter. The two stimuli from the negative control condition lacked both semantic and verbal content (−M/−L): (i) an audio clip of Paganini’s Caprice No. 24 arranged for solo flute, denoted as “Flute” hereafter; and (ii) a video consisting of continuously changing kaleidoscope images, denoted as “Kaleidoscope” hereafter. Each stimulus, lasting ∼5–7 min (see Table 2 for detailed timing information), was preceded and followed by 16 s of fixation. All the materials are available on OSF (https://osf.io/t8sz5/?view_only=aeae74892557444b80753700d3819286), except “AnimShort” (for copyright reasons).

#### Resting state scan

Ten participants additionally performed a resting state scan, used in a control analysis. Participants were instructed to close their eyes and let their mind wander but to remain awake while resting in the scanner for 5 min (the scanner lights were dimmed, and the projector was turned off).

### Data Acquisition and Preprocessing

#### Data acquisition

Whole-brain structural and functional data were collected on a whole-body 3 Tesla Siemens Trio scanner with a 32-channel head coil at the Athinoula A. Martinos Imaging Center at the McGovern Institute for Brain Research at MIT. T1-weighted structural images were collected in 176 axial slices with 1 mm isotropic voxels (repetition time [TR] = 2,530 ms; echo time [TE] = 3.48 ms). Functional BOLD data were acquired using an echo planar imaging (EPI) sequence with a 90° flip angle and using generalized auto-calibrating partially parallel aquisitions (GRAPPA) with an acceleration factor of 2; the following parameters were used: thirty-one 4-mm-thick near-axial slices acquired in an interleaved order (with 10% distance factor), with an in-plane resolution of 2.1 × 2.1 mm, field of view (FoV) in the phase encoding (A > > P) direction 200 mm and matrix size 96 × 96 mm, TR = 2,000 ms and TE = 30 ms. The first 10 s of each run were excluded to allow for steady-state magnetization.

#### Spatial preprocessing

Data preprocessing was performed with SPM12 (using default parameters, unless specified otherwise) and custom scripts in MATLAB. Preprocessing of anatomical data included normalization into a common space (Montreal Neurological Institute [MNI] template) and segmentation into probabilistic maps of the gray matter (GM), white matter (WM), and cerebro-spinal fluid (CSF). A GM mask was generated from the GM probability map and resampled to 2 mm isotropic voxels to mask the functional data. The WM and CSF maps were used as described below. Preprocessing of functional data included motion correction (realignment to the mean image using second-degree b-spline interpolation), normalization (estimated for the mean image using trilinear interpolation), resampling into 2 mm isotropic voxels, and smoothing with a 4 mm full-width, half-maximum (FWHM) Gaussian filter.

#### Temporal preprocessing

The localizer runs were high-pass filtered at 128 s. Additional preprocessing of data from the naturalistic runs was performed using the CONN toolbox (Whitfield-Gabrieli & Nieto-Castañón, 2012; https://www.nitrc.org/projects/conn) with default parameters, unless specified otherwise. BOLD signal time courses were extracted from WM and CSF. Five temporal principal components were obtained from each, as well voxel-wise averages. These were regressed out of each voxel’s time course, along with additional noise regressors, specifically, six motion parameters estimated during offline motion correction (three translations and three rotations) and their first temporal derivatives, and artifact time points (based on global signal outliers and motion).

### Modeling Localizer Data

For the localizer task, a standard mass univariate analysis was performed in SPM12 whereby a general linear model estimated the effect size of each condition in each experimental run. These effects were each modeled with a boxcar function (representing entire blocks) convolved with the canonical hemodynamic response function. The model also included first-order temporal derivatives of these effects, as well as nuisance regressors representing entire experimental runs, offline-estimated motion parameters, and time points classified as outliers (i.e., time points where scan-to-scan differences in global BOLD signal were above 5 standard deviations, or where the scan-to-scan motion was above 0.9 mm). The obtained weights were then used to compute the functional contrast of interest: sentences > nonwords.

### Participant-Specific Functional Localization of the Language Network

#### Defining functional regions of interest

Language functional regions of interest (fROIs) were defined individually for each participant based on functional contrast maps from the localizer experiments (a toolbox for this procedure is available online; https://evlab.mit.edu/funcloc/). These maps were first restricted to include only GM voxels by excluding voxels that were more likely to belong to either the WM or the CSF based on SPM’s probabilistic segmentation of the participant’s structural data, as described above.

Then, fROIs in the language network were defined using group-constrained, subject-specific localization (Fedorenko et al., 2010). For each participant, the map of the sentences > nonwords contrast was intersected with binary masks that constrained the participant-specific language network to fall within areas where activations for this contrast are likely across the population. These masks are based on a group-level representation of the contrast obtained from an independent large (*n* = 220) sample of participants. We used five such masks in the left-hemisphere, including regions in the inferior frontal gyrus, in its orbital part, in the middle frontal gyrus, in the anterior temporal lobe, and in the mid-to-posterior temporal lobe. These masks are available online (https://evlab.mit.edu/funcloc/). In each of these five masks, a participant-specific language fROI was defined as the 10% of voxels with the highest *t* values for the sentences > nonwords contrast. (Note that this contrast allows for the inclusion of voxels in a fROI that respond above baseline to the nonwords condition, as long as these responses are weaker than to sentences.)

### Critical Analysis: ISCs

#### Computing ISCs

For each participant, BOLD signal time courses recorded during each naturalistic condition were extracted from each voxel beginning 6 s following the onset of the stimulus (to exclude an initial rise in the hemodynamic response relative to fixation, which could increase ISCs). These time courses were temporally *z*-scored in each voxel and then averaged across voxels within each fROI. Then, for each fROI, participant, and condition we computed an ISC value, namely, Pearson’s moment correlation coefficient between the time course and the corresponding average time course across the remaining participants (Figure 2B; Lerner et al., 2011). ISCs were Fisher-transformed before statistical testing to improve normality (Silver & Dunlap, 1987).

We also tested whether the relatively high ISCs in +M/−L conditions could be attributed to specific high-response time points. In particular, specific scenes in the stimuli might activate the language network across participants because they elicit some linguistic representations. In other words, the tracking of the +M/−L conditions may not be due to the processing of meaningful nonverbal information but instead be driven by a few points where participants actually engage in linguistic processing. To evaluate this possibility, ISCs were recomputed after peak time points (or trough time points, as a control) of the BOLD signal were removed (peak- / trough-removed ISCs; Figure 2C). To define peaks, a mean BOLD signal was constructed for each condition by averaging the time course across participants and fROIs. Peak time points were defined as those with values greater than 1 in the *z*-normalized average time course. Similarly, trough time points were defined as time points with values lower than −1 in the *z*-normalized average time course.

#### Statistical testing

In the critical analyses, we compared ISCs across condition types using linear mixed-effects (LME) models (Barr et al., 2013), implemented in R (R Core Team, 2023). ISCs were modeled with condition type as a fixed effect and fROI, participant, and condition (stimulus) as random intercepts. The model was tested for significance in two-tailed hypothesis tests. Mixed-effects modeling is more robust to missing-at-random data (i.e., the number of stimuli per participant; see Table 1), as well as to unbalanced designs (i.e., different numbers of conditions per condition type). For all LME analyses, we coded condition type using the dummy coding scheme, with +M/−L condition type serving as a reference level, unless specified otherwise.

We additionally investigated ISCs for each +M/−L stimuli separately by treating each +M/−L stimulus as an individual condition type, which resulted in six condition types in total (+M/+L, −M/−L, AnimShort, SilentFilm, IntentShapes, and SoundEffectStory). We then compared the ISCs during each condition to the zero baseline. ISCs were modeled with condition type as a fixed effect and fROI and participant as random intercepts. Additionally, ISCs during each +M/−L condition were compared to ISCs during +M/+L and −M/−L condition types. Here, ISCs during +M/+L and −M/−L condition types were used as reference levels. *P* values are reported following false discovery rate (FDR) correction for multiple (*n* = 3; against zero baseline, +M/+L, and −M/−L) comparisons (Benjamini & Yekutieli, 2001).

Furthermore, to estimate the effect of condition type within individual regions of the language network, we modeled ISCs for each fROI separately, with condition type as a fixed effect and participant and condition as random intercepts. To test whether each +M/−L stimulus fell above baseline for each fROI, each +M/−L stimulus was recoded as a separate condition type, similar to above, and ISCs were modeled with condition type as a fixed effect and participant as random intercepts with zero baseline as a reference level. When modeling responses within individual fROIs, *p* values were FDR-corrected for multiple comparisons across fROIs (*n* = 5). To directly assess the interaction of fROIs and condition type, a second version of the model was constructed such that ISCs were modeled with fROI and fROI:condition type (interaction term) as a fixed effect and participant, and condition (stimulus) as random intercepts.

Finally, to establish whether ISCs were driven by peaks and troughs in the ISC time courses, we compared peak-removed and trough-removed ISCs for each condition against a null distribution of ISCs (*n* = 10,000), where *m* time points in the time course were pseudo-randomly removed (*m* matched the number of peak time points for each condition). These adjusted time courses were generated in MATLAB 2018a (Mathworks, 2018). The peak-removed and trough-removed ISCs were also tested against each other using a two-sample *t* test for each condition. For the latter analysis, trough-removed ISCs were adjusted so that the number of time points removed (the ones with the lowest values) matched the number of peak time points removed for each condition. *P* values were FDR-corrected for multiple comparisons across conditions (*n* = 4).

### Content Unit Analysis

To measure the degree of semantic content in a given stimulus, we quantified the number of Content Units (CUs) in each clip. We defined a CU as a unique concept (object, entity, action, property, etc.) referred to in verbal descriptions of each stimulus (Agis et al., 2016; Gallée et al., 2021). To estimate the number of CUs in each clip, we conducted an online behavioral study with an independent pool of participants.

The task was implemented as an online Qualtrics (2024) survey that presented a randomly chosen stimulus to each participant. The stimulus was cut into ∼30 s subclips, and the subclips were presented in chronological order. The splitting of stimuli into subclips was done manually to minimize abrupt cuts that would split individual events (e.g., Richmond & Zacks, 2017). The same subclips were used for all participants for a given stimulus. This task design allows each participant to watch/listen to the entire clip to understand the long-timescale structure of the stimuli, while also having a frequent opportunity to write down their descriptions before they forget the details (although fine details may still be lost in this setup, due to working memory limitations and information compression that characterizes verbal accounts). Each subclip was followed by a text box page, where participants were asked to describe the stimulus they just watched/listened to. The transition to the text box page was enabled 5 s after the clip ended. At the end of the survey, as an attention check, participants were asked two questions about the content of the stimulus they had been presented. Any given participant could participate in the task only once.

Two hundred fifty native English speakers between the ages of 19 and 77 were recruited online via the Prolific (2024) platform. Three participants were removed for failing attention checks at the end of the survey and providing responses with little meaningful content. Of the 247 remaining participants, 20 participants per stimulus were randomly selected (for a total of 200 participants; *n* = 20 × 10 stimuli) for further analyses; this was done to ensure each stimulus had the same number of responses.

Before analyzing the descriptions for the number of CUs, the following (content-irrelevant) categories of phrases were manually removed: explicitly self-referential language (“I think,” “I believe,” etc.), meta-descriptive language (“Same as before,” “In this clip,” “the camera,” “the screen,” etc.), and language regarding levels of confidence/belief states (“It appears to be,” “seems like,” etc.). The remaining portions of the descriptions were stripped of stop words (high-frequency function words) using the nltk package (Bird et al., 2009) in Python, lemmatized using the spaCy package (Version 3.4.3; Honnibal et al., 2020) in Python, and split into individual words. Words that served as subcomponents of proper nouns (e.g., “Mr. Bean,” “Elvis Presley”) were merged and counted as a single CU. Words that appeared multiple times within the response to a single subclip were treated as different CUs because the same entity could participate in multiple events within the clip. For the analysis, we only included CUs that were used by three or more participants.

To statistically compare CU counts between condition types, we used an LME to model average (across participants) subclip CU counts with condition type as a fixed effect and condition (stimulus) as a random intercept. To assess the relationship between CU counts and the ISCs across the stimuli, we further averaged the CU counts across subclips within a clip (averaging was used instead of summing in order to normalize for differences in the number of subclips per clip). First, a Pearson correlation coefficient between the average CU count and the average ISCs was computed, each condition contributing to a single data point (total 10 data points). Second, a model comparison examined whether semantic richness (as approximated by CU counts) explained additional variance in ISCs over condition type. We created two models to predict ISCs of stimuli from the three condition types: one adding normalized CU count (centered around 0) as a fixed effect to the original model and the other replacing condition type with normalized CU count for the fixed effect. The first model was compared with the original model using the likelihood ratio test. The second model was compared with the original model using AIC (Akaike information criterion) given that the two models were not nested. The above analyses were also performed using pairwise shared CU count, defined as the average number of CUs that were shared between each participant pair.

## RESULTS

### Language Regions Reliably Track Meaningful Nonverbal Stimuli

**Figure 3.**
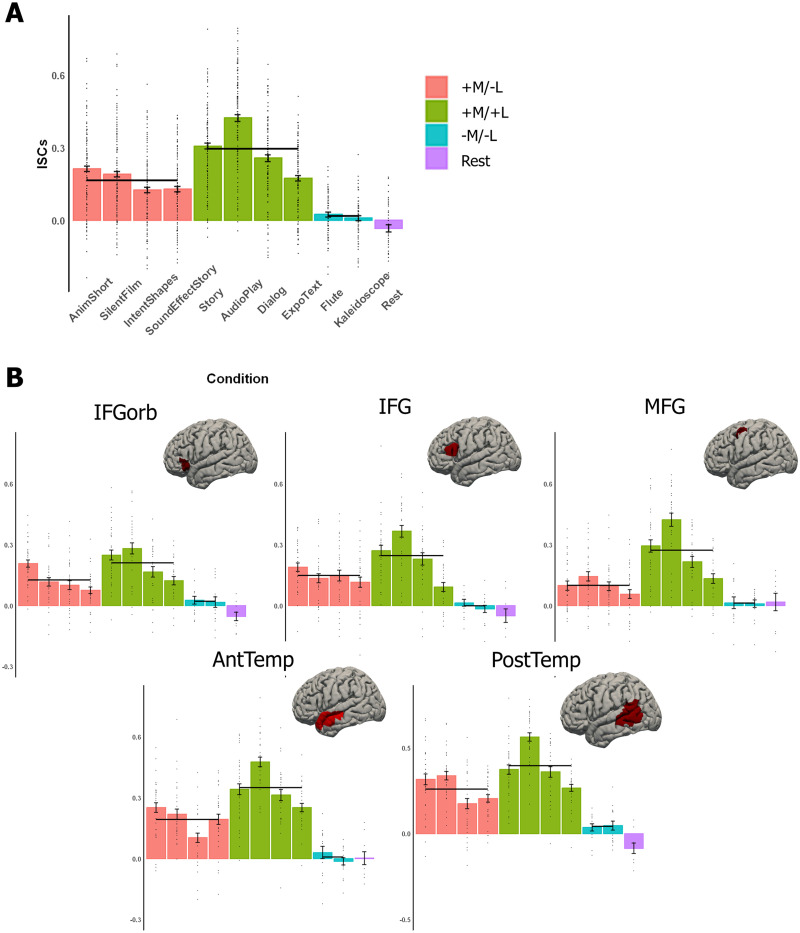
ISCs in the language network. A. Average ISCs across the five language fROIs. Here and in B, dots indicate individual participants’ data points, error bars indicate standard error of the mean by participant, and black horizontal lines indicate the mean ISC values for each of the three condition types. B. Average ISCs for each language fROI separately (on the brains, we show the parcels that were used to define the fROIs; individual fROIs comprise 10% of each parcel, as described in the text). IFGorb = inferior frontal gyrus, orbital portion; IFG = inferior frontal gyrus; MFG = middle frontal gyrus; AntTemp = anterior temporal cortex; PostTemp = posterior temporal cortex.

Meaningful nonverbal (+M/−L) conditions elicited ISCs that were significantly higher than the zero baseline (mean ISCs = 0.164, beta = 0.168, *SE* = 0.0434, *p* = 0.00151; Figure 3A). As a check, we also examined the BOLD signal during the resting state condition to ensure that spurious correlations do not arise from the data acquisition and analysis procedures. The ISCs during rest averaged across fROIs were not greater than 0 (one-tailed *t* test, *t*(9) = −1.53, *p* = 0.920), suggesting that the ISCs for the other conditions reflect stimulus-related activity.

To characterize the effect of reliable ISCs for the meaningful nonverbal conditions in greater detail, we next tested individual +M/−L conditions against the zero baseline. Each of the four +M/−L conditions elicited above-baseline ISCs (Table 3, top). The language fROIs tracked these nonverbal stimuli regardless of whether they were visual (AnimShort, SilentFilm, IntentShapes) or auditory, made up of sound effects (SoundEffectStory).

**Table 3.** Linear mixed-effect model statistics for the +M/−L conditions.

Contrast	Condition	beta	*SE*	*p* value	
+M/−L vs. 0	AnimShort	0.204	0.0281	<0.001	***
	SilentFilm	0.181	0.0282	<0.001	***
	IntentShapes	0.115	0.0282	0.00314	**
	SoundEffectStory	0.121	0.0282	0.00230	**
+M/−L vs. +M/+L	AnimShort	−0.0869	0.0125	<0.001	***
	SilentFilm	−0.110	0.0125	<0.001	***
	IntentShapes	−0.176	0.0126	<0.001	***
	SoundEffectStory	−0.170	0.0127	<0.001	***
+M/−L vs. −M/−L	AnimShort	0.203	0.0164	<0.001	***
	SilentFim	0.179	0.0164	<0.001	***
	IntentShapes	0.113	0.0164	<0.001	***
	SoundEffectStory	0.119	0.0166	<0.001	***

*Note*. For each condition (stimulus), ISCs were compared against the zero baseline, average +M/+L ISCs, and average −M/−L ISCs using LME models with condition as a fixed effect and participant and fROI as random intercepts. For each analysis, the following three values are reported: beta, standard error of the mean (SE), *p* value (FDR-corrected for multiple comparison across baselines [*n* = 3]). For *p* values, * < 0.05, ** < 0.01, *** < 0.001.

We next asked whether the recruitment of the language network by +M/−L stimuli can be observed at the level of individual fROIs (Figure 3B). Indeed, these conditions elicited ISCs that were significantly higher than the zero baseline in each of the five fROIs (Table 4). These results also held for each condition individually, except for SoundEffectStory in middle frontal gyrus (Table S2). Although the latter result may indicate some degree of functional heterogeneity among the components of the language network, overall, these analyses show that naturalistic meaningful nonverbal stimuli, including both visual and auditory stimuli, elicit reliable stimulus-related activity across the language network, as indexed by reliable ISCs.

**Table 4.** Responses to +M/−L stimuli in each fROI.

fROI	beta	*SE*	*p* value	
IFGorb	0.127	0.0278	0.00126	**
IFG	0.149	0.0353	0.00186	**
MFG	0.0976	0.0380	0.0260	*
AntTemp	0.192	0.0337	<0.001	***
PostTemp	0.262	0.0428	<0.001	***

*Note*. For each fROI, we estimated LMEs condition type as a fixed effect and participant and condition as random intercepts. For each fROI, we report the LME’s intercept value (+M/−L against 0). The following three values are reported: beta, standard error of mean (*SE*), *p* value (FDR-corrected for multiple comparison across fROIs [*n* = 5]). IFGorb = inferior frontal gyrus, orbital portion; IFG = inferior frontal gyrus; MFG = middle frontal gyrus; AntTemp = anterior temporal lobe; PostTemp = posterior temporal lobe. For *p* values, * < 0.05, ** < 0.01, *** < 0.001.

Taken together, these analyses show that naturalistic meaningful nonverbal stimuli, including both visual and auditory stimuli, elicit reliable stimulus-related activity across the language network, as indexed by reliable ISCs.

### Language Regions Track Meaningful Nonverbal Stimuli More Reliably Than Non-meaningful Stimuli, But Less Reliably Than Verbal Stimuli

We next asked how the ISCs elicited by the +M/−L conditions differ from the ISCs elicited by the other two condition types. We found that the ISCs elicited by +M/−L conditions (mean ISCs = 0.164, beta = 0.168, *SE* = 0.0434, *p* = 0.00151) that were higher than those elicited by −M/−L conditions (mean ISCs = 0.0163, beta = −0.146, *SE* = 0.0597, *p* = 0.0350) but lower than those elicited by +M/+L conditions (mean ISCs = 0.295, beta = 0.118, *SE* = 0.0483, *p* = 0.0353). This pattern also held for each individual condition (Table 3, middle and bottom rows). To test individual fROIs’ sensitivity to the three conditions of interest, we re-ran the LME model with fROI and the interaction of fROI and condition type as a fixed effect (full formula: ISCs ∼ 0 + fROI + fROI: condition type + (1 | condition) + (1 | participant)). We observed a significant effect in the +M/−L vs. +M/+L contrast in the middle frontal gyrus language fROI and in the two temporal language fROIs. Similarly, there was a significant effect in the +M/−L vs. −M/−L contrast in the IFG language fROI and in the two temporal language fROIs. Both the +M/+L > +M/−L and the +M/−L > −M/−L effects held numerically for all fROIs; Table S3, bottom row, statistics on individual fROI (Table S3, top row) are reported for completeness). In sum, although all language fROIs showed consistently stronger tracking for +M/+L over +M/−L over −M/−L, the reliability of these differences varied across fROIs. Investigating potential functional differences in ISCs across regions would require pairwise fROI comparisons in a sufficiently well-powered design to allow correction for multiple comparisons.

Taken together, these analyses show that meaningful nonverbal stimuli elicit stimulus-related activity across the language network that is stronger than that elicited by non-meaningful nonverbal stimuli (e.g., a musical piece) but weaker than that elicited by meaningful verbal stimuli (e.g., a story).

### Elevated ISCs in the +L Conditions Cannot Be Explained by the Amount of Semantic Content

**Figure 4.**
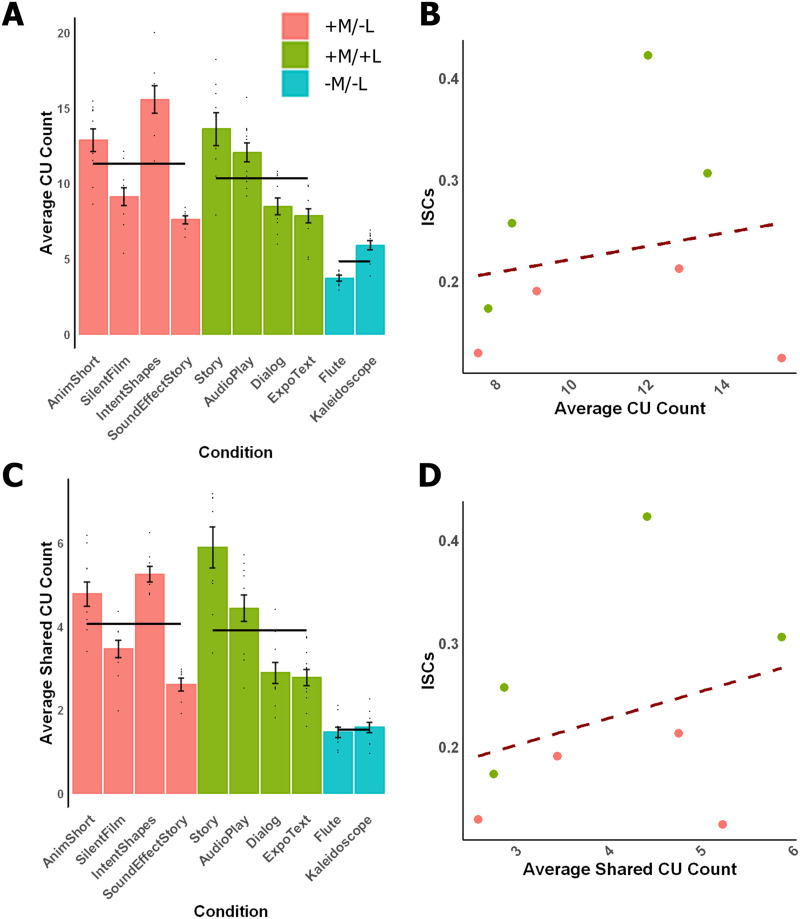
Content unit (CU) count analysis. A. Average CU count across participants. Dots indicate individual subclips that comprise each stimulus, and error bars indicate standard error of the mean by subclip. Black horizontal lines indicate the mean CU counts for each of the three condition types. B. The correlation between the average CU count and the ISC across the +M stimuli. Dashed line indicates best fit. C, D. Same as A and B, but using average shared CU count.

Does the difference in ISCs elicited by the +M/+L versus the +M/−L conditions reflect a difference in semantic richness? To test this possibility, we quantified the degree of semantic richness for each stimulus using the CU metric—the number of referenced concepts in verbal descriptions of the stimuli (Figure 4A; see Content Unit Analysis for details). As a check, we first examined the CU counts for the −M conditions. The CU count was significantly lower in the −M/−L conditions compared to both the +M/−L (beta = 6.48, *SE* = 2.19, *p* = 0.0143) and the +M/+L conditions (beta = 5.68, *SE* = 2.18, *p* = 0.0267), in line with our classification of +M versus −M stimuli.

We then tested whether the +M/+L conditions were more semantically rich compared to the +M/−L conditions. These two condition types did not differ statistically (beta = −0.800, *SE* = 1.96, *p* = 0.694; +M/−L was used as the reference level). Furthermore, the average CU count did not reliably correlate with ISC strength for the conditions in these two condition types (Figure 4B; *r* = 0.196, *p* = 0.642). To explicitly test for a contribution of CU counts to ISCs, we performed model comparisons including versus excluding CU count as a regressor. In the first model, we replaced the fixed effect of condition type with a fixed effect of normalized CU count. Although the normalized CU count had a small but reliable effect on ISCs (beta = 0.0196, *SE* = 0.00869, *p* = 0.0479), likely driven by the low CU count and low ISCs for −M stimuli, the model with condition type as the fixed effect fit the data better, as indicated by the reduction in AIC (ΔAIC = −4.79), suggesting that condition type explains ISCs better than CU counts. To test whether CU count explains unique additional variance over condition type, in a second model comparison, we added normalized CU count as a fixed effect on top of condition type. In this model, the CU count did not have a significant effect (beta = 0.0101, *SE* = 0.00785, *p* = 0.227), and a likelihood ratio test showed that adding this extra fixed effect did not significantly improve the model (*χ*^2^[1] = 1.54, *p* = 0.214), suggesting that semantic richness captured in CUs does not account for the stronger tracking of +L stimuli in the language regions.

To assess the generalizability of this result across metrics, we performed the same analysis using average pairwise shared CU count, which measures the similarity in the verbal description across participants and thus parallels (conceptually) the ISC measure for BOLD signals. This analysis also revealed no significant difference in the shared CU count between the two condition types (beta = −0.0348, *SE* = 0.823, *p* = 0.967; +M/−L reference level) and no significant correlation between the average shared CU count and the ISCs (Figure 4C and D; *r* = 0.324, *p* = 0.433). Again, the model with condition type as the fixed effect had a lower AIC (ΔAIC = −3.31). On this CU metric, the coefficient of normalized shared CU count was not significant (beta = 0.0541, *SE* = 0.197, *p* = 0.0210). Critically, adding a fixed effect of CU count on top of condition type did not significantly improve the model fit in a likelihood ratio test (*χ*^2^[1] = 2.29, *p* = 0.130), and the coefficient of CU count was not reliably different from zero (beta = 0.0295, *SE* = 0.0184, *p* = 0.141). Altogether, these analyses demonstrate that semantic richness most likely does not explain away the elevated ISCs in +L conditions.

### Language Network’s Tracking of Meaningful Nonverbal Stimuli Cannot Be Explained by the Peak Time Points

**Figure 5.**
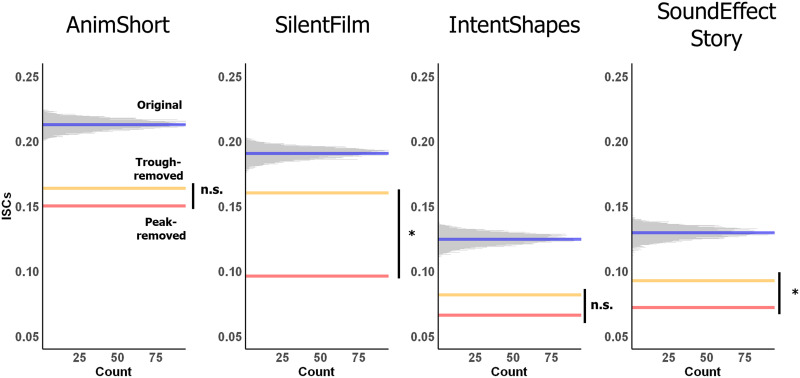
Peak-removal analysis. Peak- and trough-removed ISCs were compared against random-time-points-removed ISCs and against each other for each +M/−L condition. The histograms show the random-time-points-removed ISCs (generated 10,000 times; averaged across fROIs/participants) with bin width of 0.0001. The blue lines correspond to the true ISCs, the red lines to the peak-removed ISCs, and the yellow lines to the trough-removed ISCs. For *p* values, * < 0.05. n.s. = nonsignificant (*p* > 0.05).

In the final analysis, we asked whether the relatively high ISCs in the language network for meaningful nonverbal conditions could be explained by a small number of peak time points (when language processing is more likely to be engaged). We recomputed the ISCs after removing peak (and, for comparison, trough) time points (Figure 2C). The peak- and trough-removed ISCs were compared against ISCs computed after random time points were removed (matched in number to the peak time points removed) and against each other (Figure 5). The peak-removed ISCs were significantly lower than random-time-points-removed ISCs for each of the four +M/−L conditions (*p*s < 0.001, FDR corrected [*n* = 4]). The trough-removed ISCs were also lower than random-time-points-removed ISCs (*p*s < 0.001, FDR corrected). However, although numerically peak removal led to a larger reduction in the strength of ISCs than trough removal across conditions, this difference was only significant in one of the four conditions (SilentFilm, *t*(338) = −3.68, *p* = 0.00108; FDR corrected). Moreover, peak-removed time courses for all four +M/−L conditions still exhibited ISCs that were significantly above baseline (Table S4). These results suggest that although in some cases occasional strong responses in the language regions during the processing of meaningful nonverbal stimuli (perhaps related to activating linguistic representations) may contribute to the strength of ISCs for those conditions, these peak points alone cannot explain reliable tracking of the stimuli. In fact, in the +M/−L condition that elicited the highest ISCs in the language regions (AnimShort), peak removal and trough removal resulted in a similar reduction in ISC strength.

## DISCUSSION

A left-lateralized network of frontal and temporal areas has long been implicated in language comprehension. However, the role of this language network in nonverbal semantic processing remains debated. Using the ISC approach (Hasson et al., 2008), we examined the degree of tracking of meaningful (i.e., semantically rich) nonverbal stimuli by language areas, identified functionally in each individual (e.g., Fedorenko et al., 2010). We found that (i) all five language regions reliably track meaningful nonverbal stimuli (both visual and auditory); (ii) the degree of tracking of such stimuli is higher than for non-meaningful stimuli (e.g., music or dynamically changing kaleidoscope images) but lower than for verbal stimuli, in spite of the verbal and meaningful nonverbal stimuli being similarly semantically rich; and (iii) the tracking of meaningful nonverbal stimuli cannot be explained by the presence of occasional scenes that may elicit a strong response in the language regions (e.g., due to activating some linguistic representations).

The strong tracking of naturalistic verbal stimuli by the language network replicates several past findings (e.g., Blank & Fedorenko, 2017, 2020; Lerner et al., 2011; Wilson et al., 2008). Importantly, our analysis of CUs (Agis et al., 2016; Gallée et al., 2021) demonstrated that such elevated tracking (relative to meaningful nonverbal stimuli) is not merely a result of verbal stimuli containing higher semantic content. The CU analysis was designed to quantify the amount of semantics by capturing the subjective experience of the participants with the stimuli in fMRI. Although the verbal descriptions (elicited for our CU analysis) may miss some of the fine details of the stimuli, the fact that the CU counts match our initial classification of stimuli into meaningful (+M) and non-meaningful (−M) suggests that the CU analysis is a useful tool for quantifying the amount of semantics in a given stimulus. That said, we recognize that meaningful verbal and meaningful nonverbal stimuli (in general, or for the particular set used in the current study) may differ under other metrics of semantic content. The reliably stronger tracking of verbal than nonverbal stimuli in the language network adds to the growing body of evidence suggesting that the language network is highly selective for language processing relative to diverse nonlinguistic functions (e.g., Fedorenko et al., 2011; Ivanova et al., 2020; Monti et al., 2009; Paunov et al., 2022; Pritchett et al., 2018).

Another key question is whether different fROIs of the language network play distinct roles in processing nonverbal semantic content. Whereas +M/+L > +M/−L and +M/−L > −M/−L differences were observed at the level of the language network as a whole, only three out of five fROIs showed significance for each contrast when fROIs were analyzed individually. These differences might reflect either a lack of power or real underlying differences in sensitivity to verbal versus nonverbal meaning (Bi, 2021; Devereux et al., 2013; Visser et al., 2012; Wurm & Caramazza, 2019; Xu et al., 2016). Future work should test whether such regional specificity exists within the language network for semantic processing, but such claims would need to be supported by region-by-condition interaction statistics (Nieuwenhuis et al., 2011).

The observed difference between the meaningful verbal and meaningful nonverbal stimuli requires care in its interpretation. Lower ISCs in the +M/−L compared to the +M/+L conditions imply lower consistency in responses to these stimuli across participants, but not necessarily lower evoked responses. In other words, this result on its own does not rule out the possibility that the language network is similarly responsive to meaningful verbal and meaningful nonverbal content but that for nonverbal stimuli, participants attend to different aspects of the stimuli, which leads to lower ISCs. However, previous work argues against this possibility. Specifically, Ivanova et al. (2021) found that event pictures activate the language network to a lesser degree compared to semantically matched sentences (discussed in more detail below). Similar results were obtained using auditory meaningful nonverbal stimuli (e.g., the sound of a gunshot, followed by the sound of footsteps running away; Humphries et al., 2001), nonlinguistic computer code-based descriptions of action sequences (Ivanova et al., 2020), nonverbal theory of mind scenarios (Shain, Paunov, et al., 2023), and pictures of objects (Benn et al., 2023). These findings suggest that the more likely explanation for the lower ISCs for the meaningful nonverbal (+M/−L) stimuli is the language network’s lower degree of engagement with those stimuli rather than merely a lower degree of response consistency.

Worth a special mention is a very low (near-zero) degree of tracking by the language network of a naturalistic musical stimulus. Although a number of neuroimaging studies have now provided evidence against the role of the language network in music perception, including in the processing of music structure (e.g., Chen et al., 2023; Deen et al., 2015; Fedorenko et al., 2011; Rogalsky et al., 2011), many researchers continue to argue for overlapping mechanisms between language and music (e.g., Kunert et al., 2015; Sammler et al., 2013). The lack of stimulus-related activity in the language network (including its inferior frontal components, i.e., Broca’s area) during naturalistic listening of a musical piece provides compelling evidence that the language areas are not performing computations related to the musical stimulus, including its structure (the most common claim; e.g., Kunert et al., 2015). Future research using intrasubject correlations based on repeated exposure to the same music stimulus can further clarify whether this result is due to the lack of engagement of the language network during music listening or due to the interpretive freedom associated with music stimuli.

Our main finding—above-baseline tracking by the language network of meaningful nonverbal stimuli—is in line with recent work showing that (a) event pictures elicit a reliable response in the language network, but (b) this response is lower than that elicited by semantically matched sentences (Ivanova et al., 2021). Here, we extend these results in an important way by ruling out the possibility that the responses in the language network to visual events are task-driven. In particular, it has been suggested that when viewing visual objects and events, participants may activate words related to those stimuli in order to facilitate task performance (Greene & Fei-Fei, 2014; Trueswell & Papafragou, 2010). However, naturalistic stimuli presented under passive-viewing instructions are unlikely to lead to verbal re-coding given no obvious advantages of such re-coding. And the possibility of occasional verbal re-coding—perhaps in the parts of the stimuli that impose high processing or memory demands—is further ruled out by our peak-removal analysis, which showed that the observed ISCs are not driven by a small number of time points with elevated activity in the language network.

Together, our results suggest that the language network exhibits continuous and automatic tracking of semantic content in nonverbal stimuli. What does this tracking reflect? Data from neuropsychological investigations of patients with acquired brain damage (e.g., Chertkow et al., 1997; Colvin et al., 2019; Ivanova et al., 2021; Saygin et al., 2004; Warren & Dickey, 2021; Whitehouse et al., 1978) demonstrate that damage to the language network does not impair visual/abstract semantic processing. For example, Ivanova et al. (2021; see also Dickey & Warren, 2015) showed that some individuals with aphasia (including severe aphasia) perform well on a challenging semantic task on event pictures (judging event plausibility for events with two animate participants; e.g., a jester entertaining a king vs. a king entertaining a jester). However, it is still possible that the language network contributes to semantic processing—redundantly with other systems—without being crucial for it.

Another possibility is that the language network’s tracking of meaningful nonverbal stimuli may reflect social-cognitive processing. All but one of our meaningful nonverbal stimuli include social interactions and all stimuli include agents. It is possible to conceive of meaningful nonverbal stimuli that are not social in nature yet satisfy our operational criteria for meaningfulness: for example, a visual sequence of natural events (e.g., an avalanche or a time-lapse of changing seasons) or a visual sequence of task instructions (e.g., for furniture assembly). Such examples are less representative of the category of meaningful nonverbal stimuli, and we have chosen to focus on more prototypical instances. However, interpreting the present results in light of this choice requires caution. Indeed, in a different study using overlapping data (Paunov et al., 2022; Table S1), the language areas were shown to track verbal stimuli with social content more strongly than verbal nonsocial stimuli, and the same may hold for the nonverbal stimuli. Interdependencies between meaning in general and social content specifically are difficult to disentangle in naturalistic-cognition paradigms without sacrificing naturalness/typicality of the stimuli we experience in day-to-day life. We see the present study—and the complementary results of Paunov et al. (2022)—as establishing a less clear-cut dissociation between language and the domain of meaning (including perhaps social information specifically) than the dissociation that characterizes the relationship between language and a broad range of other domains, like music or executive functions.

Understanding what precise semantic computations or aspects of meaning drive the language network’s response to meaningful nonverbal stimuli is an important future endeavor, which can provide important clues about the relationship between linguistic and semantic processing (Bi, 2021; Xu et al., 2016). One constraint on this space of hypotheses has to do with the size of the language network’s temporal integration (or receptive) window (e.g., Lerner et al., 2011). In particular, previous work has shown that the temporal integration window of the language network is relatively short, on the order of a clause or sentence (e.g., Blank & Fedorenko, 2020; Lerner et al., 2011; Regev et al., 2022; Shain, Kean, et al., 2023). It is therefore likely that nonverbal meanings that the language system is concerned with have to do with individually described states or agent-patient interactions, but not longer-timescale representations like situation models whose construction can span long sequences of events (Johnson-Laird, 1983; Loschky et al., 2020; Zwaan & Radvansky, 1998). But whether the language network has a preference for particular kinds or aspects of semantic content remains to be determined. Future work that manipulates in more controlled ways various properties of visual or auditory nonverbal events may help uncover such preferences, including potential dissociations among the different components of the language network.

Our study provides evidence that the language network reliably tracks meaningful nonverbal stimuli. The use of naturalistic stimuli and control analyses allowed us to rule out explanations in terms of broad differences in semantic richness and task-driven linguistic re-coding. Overall, these results demonstrate a role of the language network in naturalistic nonverbal semantic processing and set the stage for future work on the precise nature and functions of its contributions.

## ACKNOWLEDGMENTS

Yotaro Sueoka and Alexander Paunov are co–first authors. Anna Ivanova and Evelina Fedorenko are co–last authors. We would like to acknowledge the Athinoula A. Martinos Imaging Center at the McGovern Institute for Brain Research at MIT, and its support team (Steve Shannon and Atsushi Takahashi). We thank former and current EvLab members (especially Olessia Jouravlev, Zach Mineroff, Brianna Pritchett, and Caitlyn Hoeflin) for help with data collection, Amaya Arcelus for help with creating the Heider & Simmel style animation, and Jeanne Gallée and Caroline Arnold for help with recording the dialog.

## FUNDING INFORMATION

Evelina Fedorenko, Simons Foundation (https://dx.doi.org/10.13039/100000893). Evelina Fedorenko, National Institute on Deafness and Other Communication Disorders (https://dx.doi.org/10.13039/100000055), Award ID: DC016607. Evelina Fedorenko, National Institute on Deafness and Other Communication Disorders (https://dx.doi.org/10.13039/100000055), Award ID: DC016950. Evelina Fedorenko, National Institute of Neurological Disorders and Stroke (https://dx.doi.org/10.13039/100000065), Award ID: NS121471. Evelina Fedorenko, McGovern Institute for Brain Research, Massachusetts Institute of Technology (https://dx.doi.org/10.13039/100019335). Evelina Fedorenko, Department of Brain and Cognitive Sciences at MIT.

## AUTHOR CONTRIBUTIONS

**Yotaro Sueoka**: Formal analysis; Investigation; Methodology; Software; Visualization; Writing – original draft; Writing – review & editing. **Alexander Paunov**: Conceptualization; Formal analysis; Investigation; Methodology; Software; Writing – review & editing. **Alyx Tanner**: Data curation; Investigation; Methodology; Software. **Idan A. Blank**: Conceptualization; Formal analysis; Investigation; Methodology; Software. **Anna Ivanova**: Formal analysis; Methodology; Project administration; Writing – review & editing. **Evelina Fedorenko**: Conceptualization; Funding acquisition; Project administration; Writing – review & editing.

## DATA AND CODE AVAILABILITY STATEMENTS

Data and code used for this study are available on OSF (https://osf.io/t8sz5/?view_only=d5081a3bf15942c38c7d48f093eac16c). A toolbox for the language localizer is also available online (https://evlab.mit.edu/funcloc/).

## Supplementary Material


