# Supplementary material for: The Language Network Reliably “Tracks” Naturalistic Meaningful Nonverbal Stimuli
Source: Neurobiol Lang (Camb). 2024 Jun 3;5(2):385–408. doi: 10.1162/nol_a_00135 (PMC11192443; doi:10.1162/nol_a_00135)
Supplement: Supplementary file 1 [file nol-5-2-385-s001.pdf]

Supplementary Data

Condition	Language-ToM	Language-Meaning
AnimShort	+ToM, -Lang	+M/-L
SilentFilm	+ToM, -Lang	+M/-L
IntentShapes	+ToM, -Lang	+M/-L
SoundEffectStory	N/A	+M/-L
Story	+ToM, +Lang	+M/+L
AudioPlay	+ToM, +Lang	+M/+L
Dialog	+ToM, +Lang	+M/+L
ExpoText	-ToM, +Lang	+M/+L
Flute	N/A	-M/-L
Kaliedoscope	N/A	-M/-L

Table S1. *Condition and condition types.* For each condition, we report the condition type classification used in Paunov et al., 2022 (Language-ToM) and the current manuscript (Lanuage-Meaning). Stimulus not used in a study is denoted as N/A.

Condition	fROI	beta	SE	p-value	
AnimShort	IFGorb	0.206	0.0215	<0.001	***
	IFG	0.188	0.0254	<0.001	***
	MFG	0.0952	0.0265	<0.001	***
	AntTemp	0.249	0.0246	<0.001	***
	PostTemp	0.314	0.0274	<0.001	***
SilentFilm	IFGorb	0.116	0.0219	<0.001	***
	IFG	0.132	0.0258	<0.001	***
	MFG	0.140	0.0268	<0.001	***
	AntTemp	0.215	0.0249	<0.001	***
	PostTemp	0.336	0.0278	<0.001	***
IntentShapes	IFGorb	0.0997	0.0219	<0.001	***
	IFG	0.147	0.0258	<0.001	***
	MFG	0.0883	0.0268	0.00112	**
	AntTemp	0.101	0.0249	<0.001	***
	PostTemp	0.174	0.0278	<0.001	***
SoundEffectStory	IFGorb	0.0748	0.0222	0.00105	**
	IFG	0.115	0.0262	<0.001	***
	MFG	0.0498	0.0272	0.0686	
	AntTemp	0.192	0.0253	<0.001	***
	PostTemp	0.205	0.0282	<0.001	***

Table S2. *LME model statistics for each condition-fROI pair.* For each fROI, ISCs were compared across conditions/fROIs with condition type as a fixed effect and participant as random intercepts (Here, each +M/-L condition are recoded into individual condition types). Zero-baseline was used as a reference. For each fROI, the following three values are reported: beta, standard error of mean (SE), p-value (FDR-corrected for multiple comparison across fROIs (n=5)). IFGorb = inferior frontal gyrus, orbital portion; IFG = inferior frontal gyrus; MFG = middle frontal gyrus; AntTemp = anterior temporal lobe; PostTemp = posterior temporal lobe. For p-values, *<0.05, **<0.01, ***<0.001.

Contrast	beta	SE	p-value	
IFGorb	0.129	0.0358	0.00309	**
IFG	0.151	0.0358	< 0.001	***
MFG	0.102	0. 0358	0.0132	*
AntTemp	0.196	0. 0358	< 0.001	***
PostTemp	0.262	0. 0358	< 0.001	***
IFGorb : +M/±L	0.0722	0.0492	0.169	
IFG : +M/±L	0.0865	0.0492	0.105	
MFG : +M/±L	0.162	0.0492	0.00653	**
AntTemp : +M/±L	0.146	0.0492	0.0122	*
PostTemp: +M/±L	0.124	0.0492	0.0271	*
IFGorb : ±M/-L	-0.102	0.0626	0.126	
IFG : ±M/-L	-0.146	0.0626	0.0356	*
MFG : ±M/-L	-0.0846	0.0626	0.199	
AntTemp : ±M/-L	-0.182	0.0626	0.0118	*
PostTemp: ±M/-L	-0.214	0.0626	0.00424	**

Table S3. *LME model statistics for fROI : condition type interactions.* ISCs were modeled with fROI and fROI:condition type interaction as a fixed effect and participant and condition as random intercepts. +M/-L is used as reference level, and +M/±L and ±M/-L refer to +M/+L > +M/-L and +M/-L > -M/-L contrasts, respectively. Note that the terms of interest are the interaction term (bottom row); individual fROI terms (top row) are reported for completeness. For each contrast, the following three values are reported: beta, standard error of mean (SE), p-value (FDR-corrected for multiple comparison across fROIs (n=5)). IFGorb = inferior frontal gyrus, orbital portion; IFG = inferior frontal gyrus; MFG = middle frontal gyrus; AntTemp = anterior temporal lobe; PostTemp = posterior temporal lobe. For p-values, *<0.05, **<0.01, ***<0.001.

Condition	beta	SE	p-value	
AnimShort	0.150	0.0233	<0.001	***
SilentFilm	0.0960	0.0234	0.00303	**
IntentShapes	0.0660	0.0234	0.0212	*
SoundEffectStory	0.0712	0.0235	0.0149	*

Table S4. *LME model statistics for the +M/-L conditions after peak timepoint removal.* For each condition (stimulus), ISCs were compared against the zero baseline, using LME models with condition as a fixed effect and participant and fROI as random intercepts. For each condition, the following three values are reported: beta, standard error of the mean (SE), p-value. For p-values, *<0.05, **<0.01, ***<0.001.
